# Transcriptome landscape of *Mycoplasma synoviae* when exposed to chicken cells

**DOI:** 10.1186/s12864-026-12748-1

**Published:** 2026-03-28

**Authors:** Duoduo Si, Shijun Bao, Lei Guo, Fei Yang, Shenghu He, Jidong Li

**Affiliations:** 1 College of Veterinary Medicine, Animal infections disease laboratory, Gansu Agricultural University, Lanzhou, China; 2 College of Animal Science and Technology, Clinical veterinary laboratory, Ningxia University, Yinchuan, China

**Keywords:** *Mycoplasma synoviae*, Chicken cells, Infection, Prokaryotic transcriptome sequencing, Transcriptome analysis

## Abstract

**Background:**

*Mycoplasma synoviae* infection in chickens is associated with development of synovitis and arthritis, leading to a decline in poultry production and significant economic repercussions. However, the mechanism of this infection remains imperfectly known.

**Results:**

Prokaryotic transcriptome sequencing was used to identify differentially expressed genes (DEGs) in *M. synoviae* when exposed to mixed cultures of chicken macrophages and chicken embryo fibroblast cells, as well as *M. synoviae* cultured in *vitro*. Of 416 DEGs, 262 are upregulated and 122 are downregulated. Ten DEGs were randomly selected and subjected to verification through quantitative reverse transcriptase PCR. The upregulated DEGs exhibited significant enrichment in various Gene Ontology (GO) terms, notably transmembrane transport, transporter activity, and membrane protein complex. Additionally, these DEGs were enriched in Kyoto Encyclopedia of Genes and Genomes (KEGG) pathways, including ABC transporters, quorum sensing, aminoacyl-tRNA biosynthesis, DNA replication, and the bacterial secretion system. The 5’ untranslated region, transcription starting point and noncoding small RNA (sRNA) were also revealed, thirty-one upregulated genes were predicted to interact with sRNA NZ_CP011096_predRNA9.

**Conclusion:**

These findings revealed the expression of *M. synoviae* transcripts when exposed, which beneficial for the treatment of *M. synoviae’s* target proteins study, will also help to enhance understanding of mechanisms underlying *M. synoviae* infection.

**Supplementary Information:**

The online version contains supplementary material available at 10.1186/s12864-026-12748-1.

## Background

*Mycoplasma synoviae* causes acute and chronic infections in chickens that produce exudative tendinitis, synovitis and airsacculitis, leading to carcass condemnation at processing plants [1]. *M. synoviae* can inflict tissue damage, leading to inflammatory cell infiltration, and able to invade host cells such as chicken erythrocytes, chondroblasts, and embryonic cells in *vitro*. Specific strains, like *M. synoviae* ATCC 25,204 WVU 1853 strain used in this study, is noted for a higher propensity to induce synovitis and exhibit strong erythrocyte invasion capabilities [2, 3].

Extensive research has delineated the host’s transcriptional and immunological landscape post-infection. Infection of synovial sheath cells and chondrocytes with *M. synoviae* triggers the upregulation of a suite of host genes. This includes pro-inflammatory cytokines, chemokines, pattern recognition receptors, catabolic enzymes, extracellular matrix components, and pathogenicity-related proteins [4, 5]. Consequently, *M. synoviae* infection induces apoptosis in chicken chondrocytes, leading to production of nitric oxide, activation of caspase 3, and inactivation of mitochondria [6]. Collectively, these studies paint a detailed picture of host’s pathological and defensive reactions to *M. synoviae* invasion. In stark contrast, the adaptive transcriptional response of *M. synoviae* itself during host cell interaction remains poorly understood. Previous research has primarily focused on host-centric changes or the expression of a limited number of presumed virulence factors in *M. synoviae*, such as C-terminal haemagglutinin protein, cysteine protease, neuraminidase, and putative nuclease [5].The absence of a cell wall in *M. synoviae* highlights the importance of membrane proteins in its pathogenesis, making them key intervention targets. However, there is a significant gap in research on the pathogen’s survival strategies and gene expression changes when interacting with host cells [7]. A comprehensive, unbiased transcriptomic profile of *M. synoviae* during active host cell engagement is missing. This gap hinders a holistic understanding of the pathogen-host interface and the identification of novel bacterial factors essential for colonization and persistence.

To address this critical knowledge gap, this study aims to characterize the global transcriptional response of *M. synoviae* during exposure to host cells. We utilized a co-culture model employing two mature chicken cell lines, chicken macrophage HD-11 cells and chicken fibroblast DF-1 cells, which are well-established for studying poultry pathogens [8–11]. Then, we perform a comprehensive transcriptome analysis of *M. synoviae* using RNA-seq technology. *M. synoviae* gene and transcription element structure were revealed, DEGs of *M. synoviae* during host cell attack were analyzed, DEGs biological functions were visualized. Following exposure, we performed a comprehensive transcriptome analysis of *M. synoviae* using RNA-seq technology. Our objectives were to: (1) delineate the gene and transcription element structure of *M. synoviae*, (2) identify and analyze DEGs during host cell attack, and (3) visualize and interpret the biological functions of these DEGs. The findings from this study are intended to establish a foundational resource for elucidating the molecular mechanisms that enable *M. synoviae* infection and survival within host cells, ultimately informing future strategies for disease control.

## Materials and methods

### Infection experiments

The *M. synoviae* ATCC 25,204 strain was cultured in Frey liquid medium. DF-1 and HD-11 cells (Shanghai Institute of Biochemistry and Cell Biology, Shanghai) were cultured in 10% Fetal Bovine Serum (FBS) (Gibco, USA), 90% DMEM (Gibco, USA) complete cell medium until reaching the logarithmic growth phase. Cells were then seeded in 6-well plates at a 1:1 (10^5^:10^5^) ratio, after which monolayers were infected with *M. synoviae* during the mid-exponential growth phase. Infection was performed by resuspending the bacterium in DMEM at a multiplicity of infection of 100. A triplicate parallel experiment was conducted, named MS-Host1, MS-Host2, and MS-Host3. Three control samples were cultured in the modified Frey-style medium for the same duration as *M. synoviae* under identical conditions, which named MS-1, MS-2 and MS-3.

### Sample preparation for RNA isolation

After 8-h coincubation at 37 °C in a 5% CO2 incubator, supernatants were collected, host cells were washed three times with sterile phosphate buffered saline (PBS). Washing fractions were combined with supernatants and the resulting mixture centrifuged at 5000 × g for 20 min at 4 °C. The obtained pellet was used for RNA extraction using TRIzol reagent (Thermo Fisher Scientific, USA) following standard procedures.

### Total RNA isolation and preparation of copy DNA (cDNA) libraries for sequencing

Total RNA was extracted separately for each sample using the TRIzol method [12]. RNA quality was analyzed by Agilent Bioanalyzer, and its concentration and purity was analyzed by NanoDrop spectrophotometer (IMPLEN, CA, USA). The RiboZero rRNA Removal Kit Bacteria (Illumina, San Diego, CA, USA) was used to deplete ribosomal RNA, and remainding DNA was eliminated from RNA samples by treatment with DNase I (TURBO DNase I, Ambion). RNA quality was verified (Supplementary file 1) before constructing the library using the dUTP strand-specific method. Subsequently, the six samples were sequenced by Gene Denovo Biotechnology Co., Ltd (Guangzhou, China) via the Illumina HiSeq 4000 platform.

### RNA sequence data processing

For high-quality clean reads, fastp (version 0.18.0) was used as an additional filter to ensure accuracy of analysis outcomes (fastp, https://github.com/OpenGene/fastp) [13]. Bowtie2 (version 2.2.8) was used to compare clean reads with the complete ribosomal RNA sequence of *M. synoviae* and chicken to eliminate reads that aligned with the upper ribosome. Following ribosomal-read filtering, Bowtie2 was used to align them with the reference genome ((ASM96976v1; GCF_000969765.1) from the NCBI RefSeq database, https://www.ncbi.nlm.nih.gov/nuccore/NZ_CP011096.1) [14]. After performing saturation and randomness analysis, sequence results were mapped to the reference genome. Gene coverage statistics were obtained, and Fragments Per Kilobase of Transcript per Million Mapped Reads (FPKM) was applied to calculate expression levels. A comparison was made between expression abundance distributions of each sample [15]. Principal component analysis (PCA) was used to examine expression results of known genes in each sample, alongside the Pearson correlation coefficient. These approaches aimed to assess repeatability among samples and identify and eliminate outliers.

### DEGs analysis

Analysis of gene expression variations between groups was conducted using edgeR software [16], while DEGs were identified based on criteria of FDR < 0.05 and |log2Foldchange| > 1, and counts > 10. The GO database (http://geneontology.org/), serves biological process, molecular function, and cellular component annotation classifications [17, 18]. GO enrichment of DEGs were performed using the GO database, and the number of genes corresponding to each term was determined to generate a comprehensive gene list and gene count for a specific GO function. A hypergeometric test was then used to identify GO entries that exhibited significant enrichment in DEGs when compared with the overall motif group background, with q values < 0.05 used as a threshold. The KEGG database (https://www.genome.jp/kegg/pathway.html) serves as the primary public repository for pathway information [19]. The significance of pathway enrichment analysis was determined using the KEGG Pathway database; hypergeometric tests were used to identify pathways that exhibited significant enrichment in DEGs relative to the background of the entire genome; and FDR ≤ 0.05 was used as a threshold. To visualize metabolic pathways and access comprehensive organism-wide metabolic pathway information, the iPath3.0 application (http //pathways.embl.de) was used [20], data used for this analysis are listed in Supplementary Table 1.

### Other transcripts identification and analysis

Rockhopper software [21] (version 2.0.3) was used to predict transcription start and stop sites. Operons were also predicted by Rockhopper to better understand *M. synoviae* functionality. RBS-finder (version 2.0.3) (with a specified ribosome binding site region length of 20 bps) was used to predict the Shine Dalgarno sequence for the 5’ untranslated region (5’-UTR) of all genes [22].

Newly discovered transcripts (identified by Rockhopper) were subjected to a comparative analysis and annotation using the non-redundant database. Transcripts that remained unannotated were considered to be potential sRNA candidates. RNAfold software (version 2.6.4) [23] was used to predict the secondary structure of these candidate sRNA molecules, followed by a screening process to identify any differential sRNA. The interaction between differentially expressed sRNA and mRNA was predicted by CopraRNA (http://rna.informatik.unifreiburg.de/CopraRNA/Input.jsp) with default parameters. A Circos diagram was generated to visualize gene structure, where in the outermost ring (green) represents the chromosomes in 1Kbp increments, the secondary inner circle (purple for the chain of justice, light purple for the antisense chain) represents the operand subregion; and the innermost circle is dedicated to each gene (light blue, known genes; yellow, new predictor genes; and red, sRNA).

### qRT-PCR validation

Ten upregulated and downregulated genes were randomly chosen for transcriptome data validation via qRT-PCR. Genes GAPDH was used as reference genes based on the stability of its expression during our studies. Primers are listed in Table 1. Complementary DNA (cDNA) was synthesized using total RNA isolated from high-throughput sequencing experiments with a HiScript III 1st Strand cDNA Synthesis Kit (+ gDNA wiper) (Vazyme, China) and ChamQ Universal SYBR qPCR Master Mix (Vazyme, China). The 2^−ΔΔCt^ method was used, with GAPDH as an internal control for qRT-PCR results analysis. Three independent replicate experiments were performed.

**Table 1:** Primers for qPCR

Gene ID	Primer	Primer sequence (5’-3’)	PCR fragment (bp) size
VY93_RS01875	Forward	TTCAGGCGGGCAGCTTCAAAG	87
	Reverse	GATGCGATAGGCTCATCGGCTAC	
VY93_RS03640	Forward	TGAATCGTTGCTGCCAAATGAAG	77
	Reverse	CTTGCATGTGGTCAAAACCTAAAG	
VY93_RS03220	Forward	AGGCTTTGTTAAAAACTATAATGGCA	106
	Reverse	TGAAGATTGATTTTCTGCATGTTCA	
VY93_RS02985	Forward	TGCTTTCCAGGATCGCTTGATG	93
	Reverse	TTTCGCAACAGCTTGGATTTGAG	
VY93_RS01625	Forward	AAGCCGAAAACATCAAGCAAGG	150
	Reverse	GCCCTACTCCTCCAGGAACTG	
VY93_RS01160	Forward	GCTCCAGCTCCAGCAGAAAGTG	105
	Reverse	TGCTGCTTCTTCTGTTCCTGTTGC	
VY93_RS01090	Forward	GCGAGGCTCAAGCAAGACTTAGAG	110
	Reverse	ATCAGCGTTAGCCATTCCAAGACC	
VY93_RS00870	Forward	ACCGAATCAGCTTTTGCCGATGG	124
	Reverse	GCCTTTCGTGCATCATGTTAAGCC	
VY93_RS00750	Forward	TCATAAGCTTGCCTACGATAAAGC	129
	Reverse	TCTGAACGCGATCTTTGAGT	
VY93_RS00125	Forward	AGGGCTTGCCGCTCAAATTGG	79
	Reverse	CACAGTAGCCGCTACGCCTTTC	

## Results

### Quality control and alignment

**Fig. 1:**
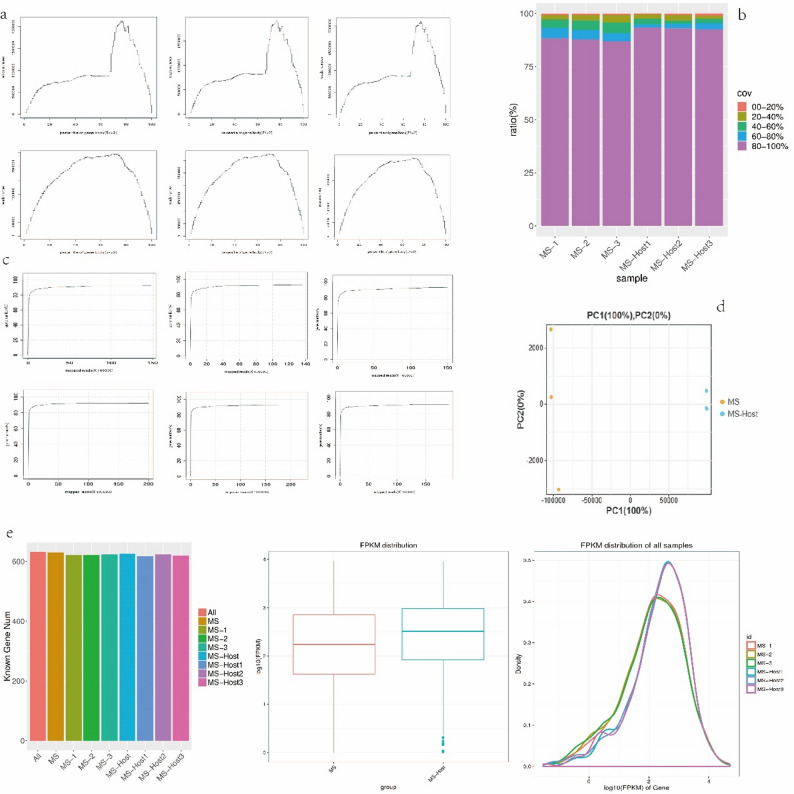
Quality control and alignment. Distribution of reads in each sample (**a**) number of reads, The first row is the reads number distribution of MS-1, MS-2 and MS-3, and the second row is the reads number distribution of MS-Host1, MS-Host2 and MS-Host3, and (**b**) percentage. **c** Sequencing saturation analysis. The first row is the Sequencing saturation diagram of MS-1, MS-2 and MS-3. The second row is the Sequencing saturation diagram of MS-Host1, MS-Host2 and MS-Host3. **d** PCA analysis based on expression of known genes in each sample. **e** Statistical analysis of genes. The abscissa is log10 (FPKM), and the ordinate is gene density. Each color in the plot represents a sample, and the sum of all probabilities is 1 (i.e., the area of each region is 1), the peak of the density curve represents the region with the highest concentration of gene expression throughout a sample

Sequence data in 6 libraries were compiled (Table 2). Each sample yielded a mean number of 20,478,044 filtered clean reads, with 88.43%–89.88% of them successfully mapped to the reference genome. A total of 632 genes were detected, accounting for 93.91% of the reference genome, with 617–624 genes (91.68%–92.72% of all identified genes) identified per sample (Fig. 1a, b). The sequencing saturation curve exhibited a gradual flattening trend, ultimately reaching a saturation standard (Fig. 1c). Principal components analysis (PCA) results indicate that the sum of PC1 and PC2 was equal to 1, with PC1 accounting for 100% of the variation and PC2 accounting for 0%, thus demonstrating a robust grouping outcome (Fig. 1d). Six samples were compared to more than 600 known genes (Fig. 1e). The FPKM distribution boxplot revealed the presence of outliers in the treatment group, with the median value of the treatment group surpassing that of the control group, both falling within the range of 2–2.5 (Fig. 1f). The distribution of the three curves in both the treatment and control groups exhibited a high degree of consistency. Specifically, the peak of the sample curves in the control group was approximately 0.4, with log10 (FPKM) values of most genes falling within the range of 1–3. Similarly, the sample curve peak in the treatment group was approximately 0.5, and the log10 (FPKM) values of most genes also fell within the range of 1–3 (Fig. 1g).

**Table 2:** Reads filter information statistics

Clean Reads Num (%)	Read Length	Adapter (%)	Low Quality (%)	PolyA (%)	*N* (%)
22,179,992 (99.48%)	150 + 150	17,846 (0.08%)	194,860 (0.44%)	0 (0%)	4 (0.0%)
20,478,044 (99.55%)	150 + 150	16,026 (0.08%)	154,516 (0.38%)	0 (0%)	2 (0.0%)
22,557,826 (99.39%)	150 + 150	22,976 (0.1%)	231,628 (0.51%)	0 (0%)	0 (0.0%)
67,419,842 (99.81%)	150 + 150	3942 (0.01%)	253,480 (0.19%)	0 (0%)	984 (0.0%)
70,353,274 (99.79%)	150 + 150	5544 (0.01%)	276,588 (0.2%)	0 (0%)	890 (0.0%)
66,329,148 (99.82%)	150 + 150	5398 (0.01%)	233,248 (0.18%)	0 (0%)	864 (0.0%)

### Visual analysis of DEGs between host cells-challenged M. synoviae and vitro-cultured M. synoviae

**Fig. 2:**
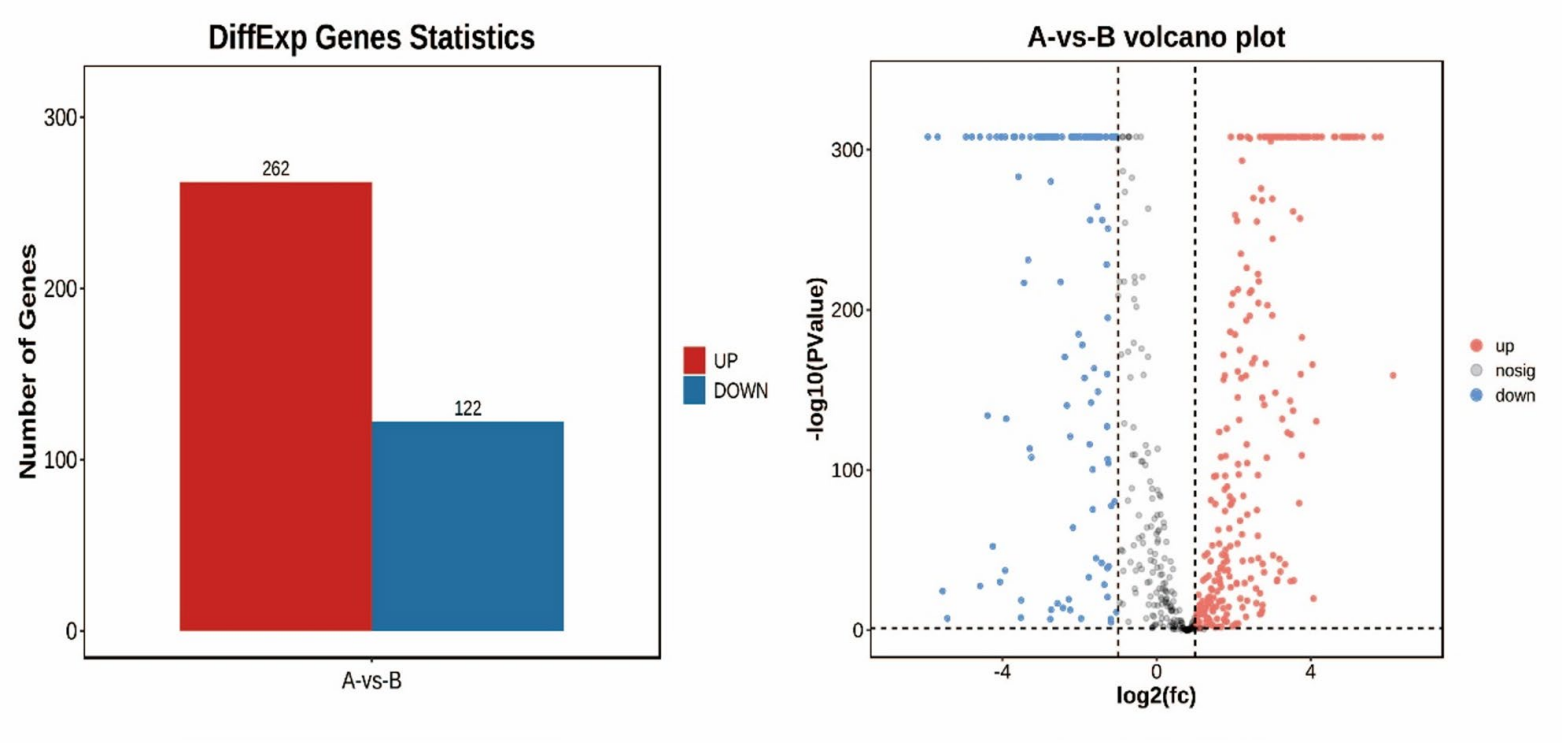
DEG visual analysis. **a** Histogram of DEGs between host cells-challenged *M. synoviae* and *vitro*-cultured *M. synoviae*. The abscissa represents the group name, and the ordinate represents the gene number. **b** Volcano plot of DEGs between host cells-challenged *M. synoviae* and *vitro*-cultured *M. synoviae*. Dot color: significantly upregulated (red), and downregulated (blue) genes; grey, non-significant genes

Upon exposure to host cells, a total of 262 genes were upregulated and 122 were downregulated (Fig. 2a). The overall distribution of DEGs was determined using volcano plots (Fig. 2b). The expressions of frequently maily studied genes which coding cysteine protease, neuraminidase and thermonuclease family protein were up-regulated (*P* < 0.05). Most coding haemagglutinin genes were significantly down-regulated (*P* < 0.05) (Table 3).

**Table 3:** Genes expression assessment which reported by Cizelj

Gene symbol	log2(FC)		Encoding protein function
cysP	VY93_RS03235	1.912381575	C1 family peptidase
	VY93_RS03855	3.11838375	thermonuclease family protein
rsuA	VY93_RS02245	1.823238312	pseudouridine synthase
nanH	VY93_RS01145	3.950062108	sialidase family protein
vlhA	VY93_RS01155	2.2862	hemagglutinin
	VY93_RS01305	-4.3862	
	VY93_RS01440	-4.5862	
	VY93_RS04075	-3.3353	
	VY93_RS04265	-2.721	
	VY93_RS04120	-3.4972	
	VY93_RS01250	-4.9483	
	VY93_RS01460	-5.9369	
	VY93_RS01280	-4.5828	
	VY93_RS01450	-1.2726	
	VY93_RS01270	-3.5141	
	VY93_RS04325	-2.5717	
	VY93_RS01465	-3.9272	

### GO and KEGG functional analysis of DEGs

**Fig. 3:**
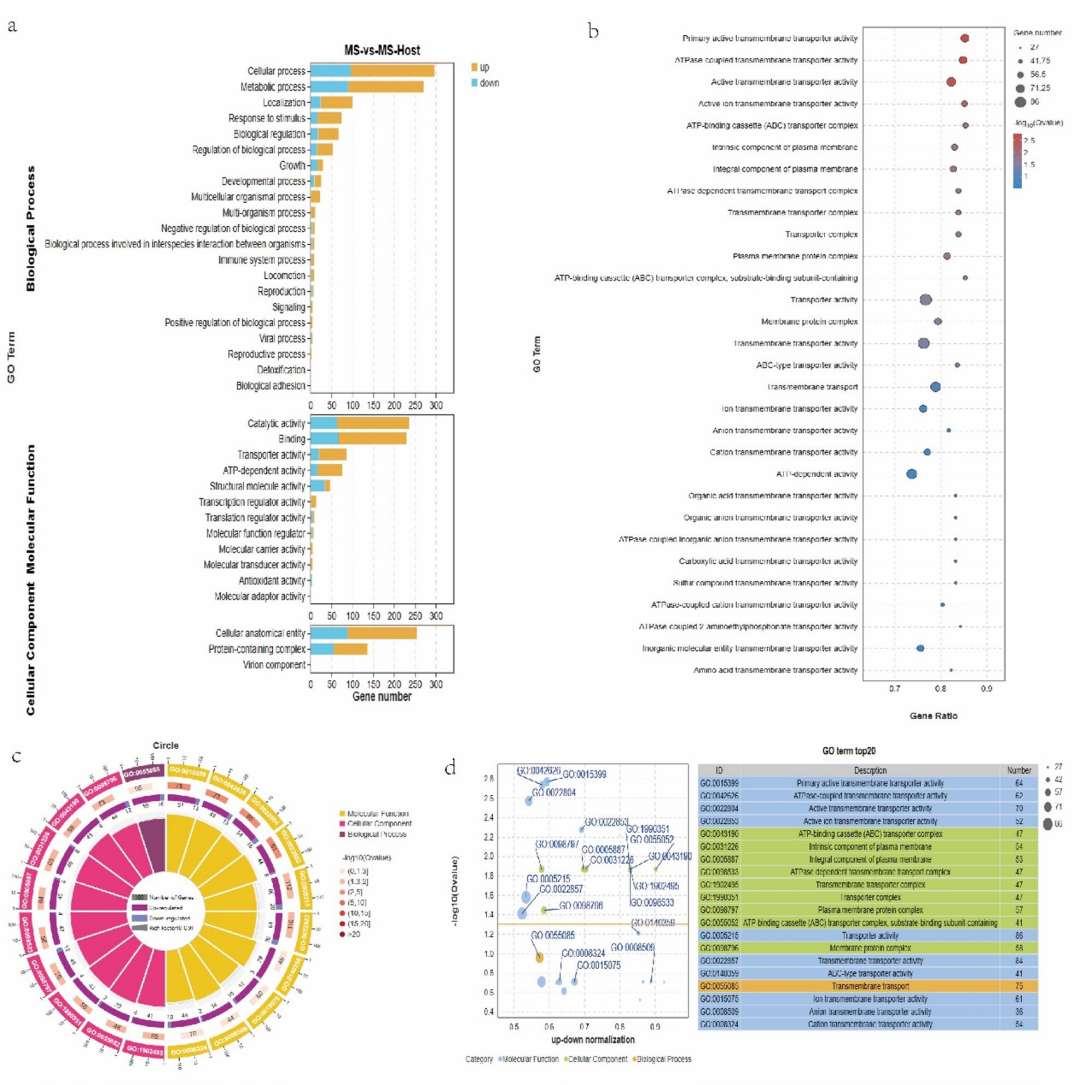
DEGs GO functional annotation. **a** Categorize secondary of DEGs GO functional annotation. The abscissa represents genes number, and the ordinate represents GO terms. Column color: orange, upregulated genes; blue, downregulated genes. **b** Bubble of DEG GO functional enrichment. The abscissa represents the gene ratio (enrichment ratio = sample number/background number) and the ordinate, the names of GO terms. Bubble size represents gene number; different color depths represent −log10 (Qvalue). The upper left corner provides a description of the GO classification. **c** Circle of DEGs GO functional enrichment. Circles: (1) GO name; (2) numbers of background genes, with color shade indicating the -log (Qvalue); (3) numbers of up- or downregulated genes; and (4) rich factor, calculated by dividing the fg_num (Up+Down num) by the bg_num. **d** Z-score bubble of DEG GO functional enrichment. The abscissa represents the up–down normalization, and the ordinate the − log (Qvalue). A higher value indicates a smaller Q/P-value, and greater significance of the corresponding bubble. The up–down normalization, quantifies the difference between proportions of up- and downregulated genes among all DEGs. A higher value on the right side of the axis indicates a larger disparity between the enrichment of up- and downregulated genes in the GO term, with a greater number of upregulated genes. The disparity between downregulated genes enriched in a given GO term compared to upregulated genes increases as one moves to the left of the specified value, with a higher count of downregulated genes. The magnitude of the bubble corresponds to the number of enriched genes, with larger bubbles indicating more enriched genes. The orange horizontal line denotes the threshold of -log (Qvalue), and bubbles positioned above this line indicate significant GO terms

The secondary categorization histogram revealed an enrichment of 21 GO terms in biological processes, 3 in cellular components, and 12 in molecular functions. Most genes enriched in biological processes were associated with cellular processes and metabolic processes, those enriched in molecular functions were associated with catalytic activity and binding, and those enriched in cellular components were mostly associated with cellular anatomical entity (Fig. 3a). The GO Enrichment Analysis Bar Chart displayed the 30 most significant GO terms, of which transporter, transmembrane transporter, primary active transmembrane transporter, ATPase-coupled transmembrane transporter, and active transmembrane transporter activities were the five most-enriched GO terms (Fig. 3b).

In the circle chart, the 20 most-significant GO terms are presented in a hierarchical manner, *P* < 0.05. The top three identified GO terms were transmembrane transport (GO: 0055085), molecular functions transporter activity (GO: 0005215), and cellular components membrane protein complex (GO: 0098796), *P* < 0.05 (Fig. 3c).

The Z-score bubble chart illustrates the prominently enriched GO terms. Because the value of up–down normalization is positive, the number of upregulated genes contained in the first 20 GO terms is greater than the number of downregulated genes, *P* < 0.05 (Fig. 3d).

**Fig. 4:**
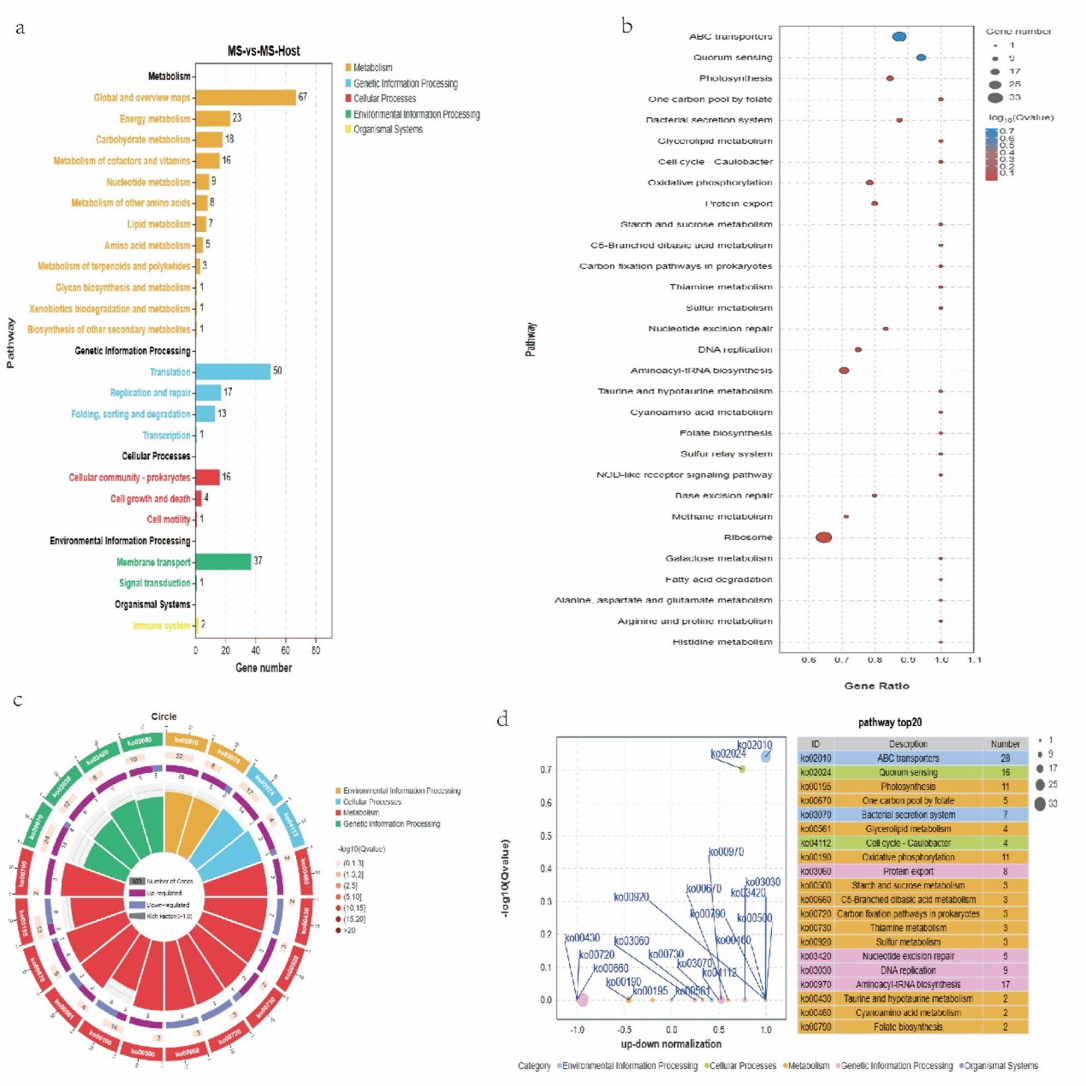
KEGG functional enrichment analysis. **a** Numbers of KEGG functionally enriched up- and downregulated genes. The abscissa represents the number of enriched genes, and the ordinate the pathway terms. **b** Bubble of keg enrichment of DEGs. The abscissa represents the gene ratio, and the ordinate the pathways. **c** Circle of KEGG enrichment of DEGs. **d** Z-score bubble of DEGs KEGG enrichment. The abscissa represents the up–down normalization, and the ordinate represents the − log (Qvalue)

Based on KEGG pathway analysis, all DEGs were predominantly enriched in five class A pathways: metabolism, genetic information processing, cellular processes, environmental information processing, and organismal systems. Additionally, 22 class B pathways were identified (each column represents a specific class B pathway). In total, 67 genes were enriched to metabolism global and overview maps, 50 to genetic information processing translation, and 37 to environmental information processing membrane transport (Fig. 4a). Bubble chart results further demonstrate significant enrichment of genes in the ABC transporters and ribosome pathways, *P* < 0.05 (Fig. 4b).

In the circle chart, the 20 most significant pathways are presented in a hierarchical manner. Notably, DEGs are enriched in specific pathways (*P* < 0.05), such as ABC transporters (ko02010), quorum sensing (ko02024), aminoacyl-tRNA biosynthesis (ko00970), photosynthesis (ko00195), and oxidative phosphorylation (ko00190) (Fig. 4c). According to the enrichment difference bubble chart, among the top 20 KEGG pathways with a significance level of *P* < 0.05, six pathways (ko00720, ko00190, ko00195, ko00430, ko00660, and ko00970) exhibit a greater number of downregulated genes compared to upregulated genes. The ko00561 pathway shows an equal enrichment of both upregulated and downregulated genes. In the remaining 13 pathways, particularly ABC transporters (ko02010), quorum sensing (ko02024), aminoacyl-tRNA biosynthesis (ko00970), DNA replication (ko03030), and the bacterial secretion system (ko03070), there is a notable enrichment of upregulated genes, with their numbers surpassing those of downregulated genes (Fig. 4d).

### iPath analysis of DEGs

**Fig. 5:**
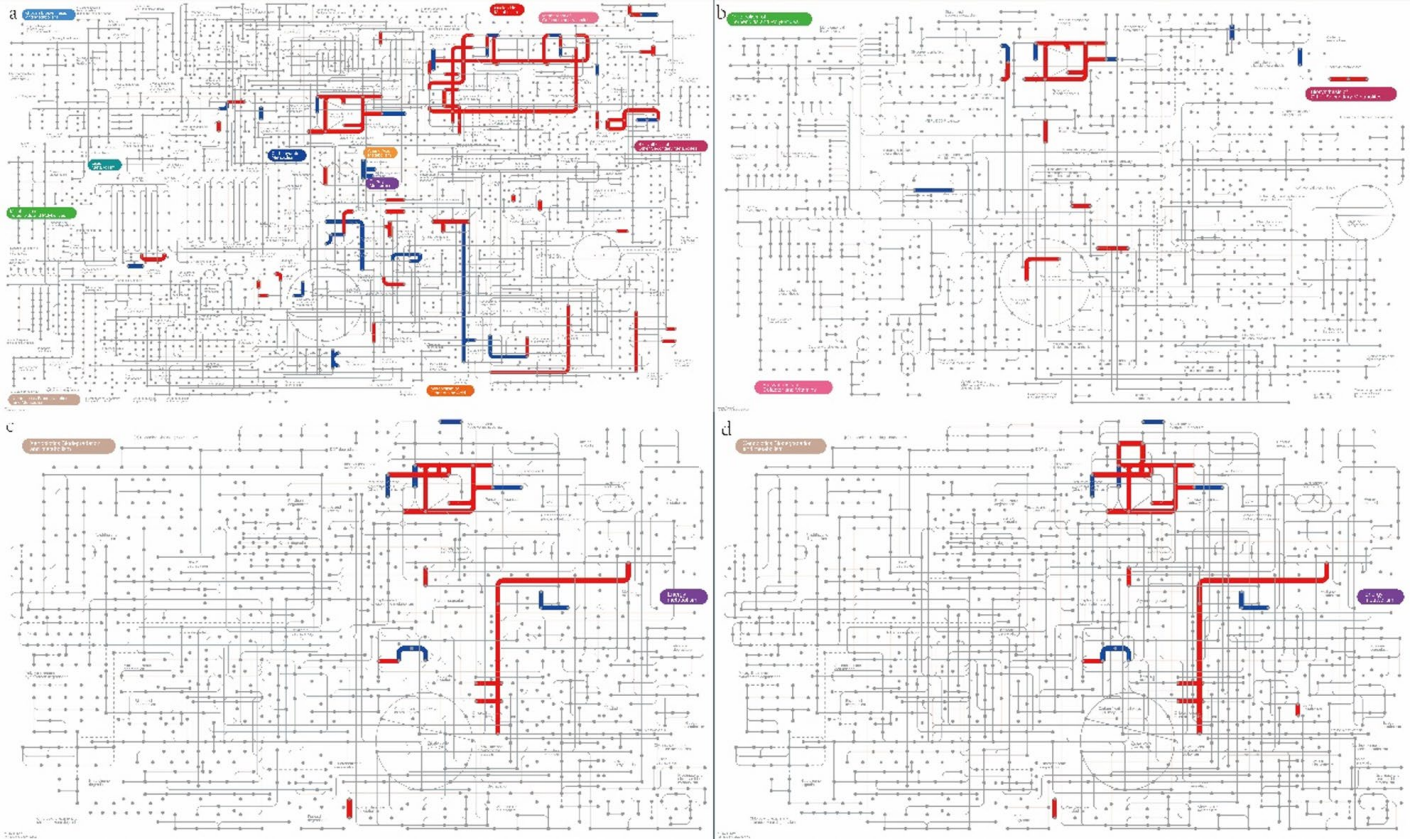
DEGs iPath analysis. Information on (**a**) metabolic pathways, (**b**) secondary metabolite biosynthesis, (**c**) multiple antibiotic synthesis, and (**d**) microbial metabolic pathways in multiple environments mapped using iPath3.0. Line colors: red, pathway affected by upregulated genes; blue, pathway affected by downregulated genes

DEG metabolic information was visualized using iPath3.0 (Fig. 5). Various pathways are included in metabolic pathways (Fig. 5a), among which aldehyde dehydrogenase (K00128) has the most hits (Supplementary Table 2a). In secondary metabolite biosynthesis (Fig. 5b), transketolase (K00615) had four hits (Supplementary Table 2b). Transketolase (K00615) was also ranked top in numbers of hits (Supplementary Table 2c) in multiple antibiotic synthesis (Fig. 5c). Fourteen pathways occurred in the microbial metabolic pathways in diverse environments module (Fig. 5d), where transketolase (K00615) had six hits (Supplementary Table 2d).

### Identification analysis of other transcripts

**Fig. 6:**
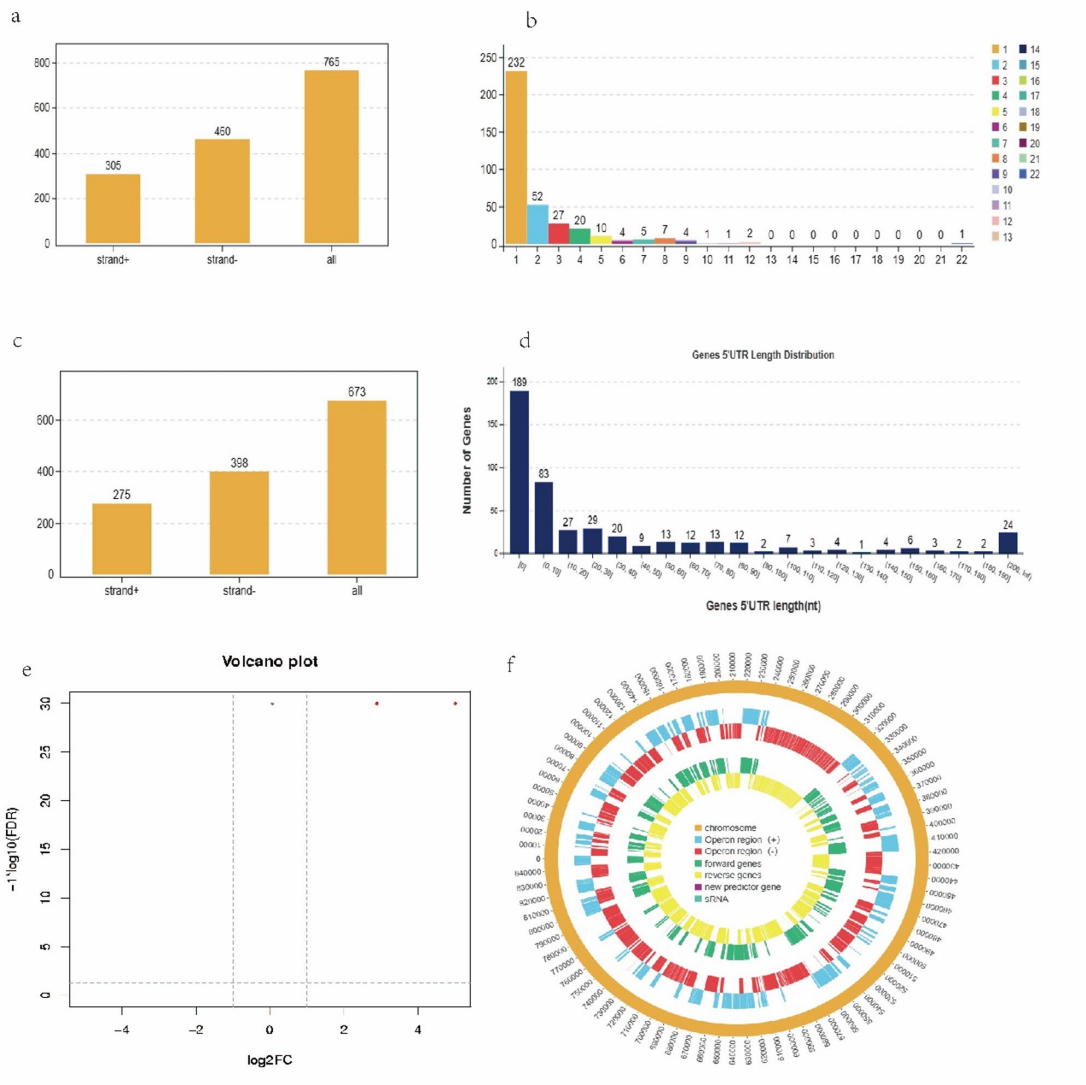
Other transcripts analysis. **a** Transcription start site analysis. **b** Operon analysis. The abscissa represents the number of genes, and the ordinate the number of operons. **c** 5’ UTR number statistics. **d** untranslated region (UTR) analysis. The abscissa represents the length of the 5’ UTR region and the ordinate the number of genes. **e** Volcano plot of sRNA differential expression. Dot colors: red, significantly upregulated genes; black, non-significant genes. **f** Visualization of gene structure

Analysis of genetic structure revealed 765 transcription start sites, with 305 located on the positive strand and 460 on the negative strand (Fig. 6a). Additionally, there were 232 operons regulate expression of 1 gene, separately. Operon_336 regulate expression of 22 genes (Fig. 6b, Supplementary Table 3). Examination of the UTR indicated that 189 genes lacked a 5’ UTR region, while 24 genes had a length exceeding 200 nucleotides (nt) (Fig. 6d). Among these, 275 were located on the positive strand and 398 on the negative strand (Fig. 6c). Three size of sRNAs range in 50 ~ 500 nt were identified (NZ_CP011096_predRNA2, 482 nt, NZ_CP011096_predRNA9, 289 nt and NZ_CP011096_predRNA10, 134 nt), sequences of three sRNA were listed in Supplementary file 1. Three sRNAs were located in coding strand of the *M. synoviae* genome, NZ_CP011096_predRNA2 rang from 231,097 to 231,578, NZ_CP011096_predRNA9 rang from 746,869 to 747,157, NZ_CP011096_predRNA10 rang from to 747,789 to 747,922. NZ_CP011096_predRNA2 and NZ_CP011096_predRNA9 were significantly upregulated (Fig. 6e). The above three elements are visualized in the specific location of the genome (Fig. 6f). Thirty-nine upregulated genes were predicted to interact with NZ_CP011096_predRNA2, among them four genes coding ABC transporter proteins (Supplementary Table 4a). Thirty-one upregulated genes were predicted to interact with NZ_CP011096_predRNA9 (Supplementary Table 4b).

### qRT-PCR validation

**Fig. 7:**
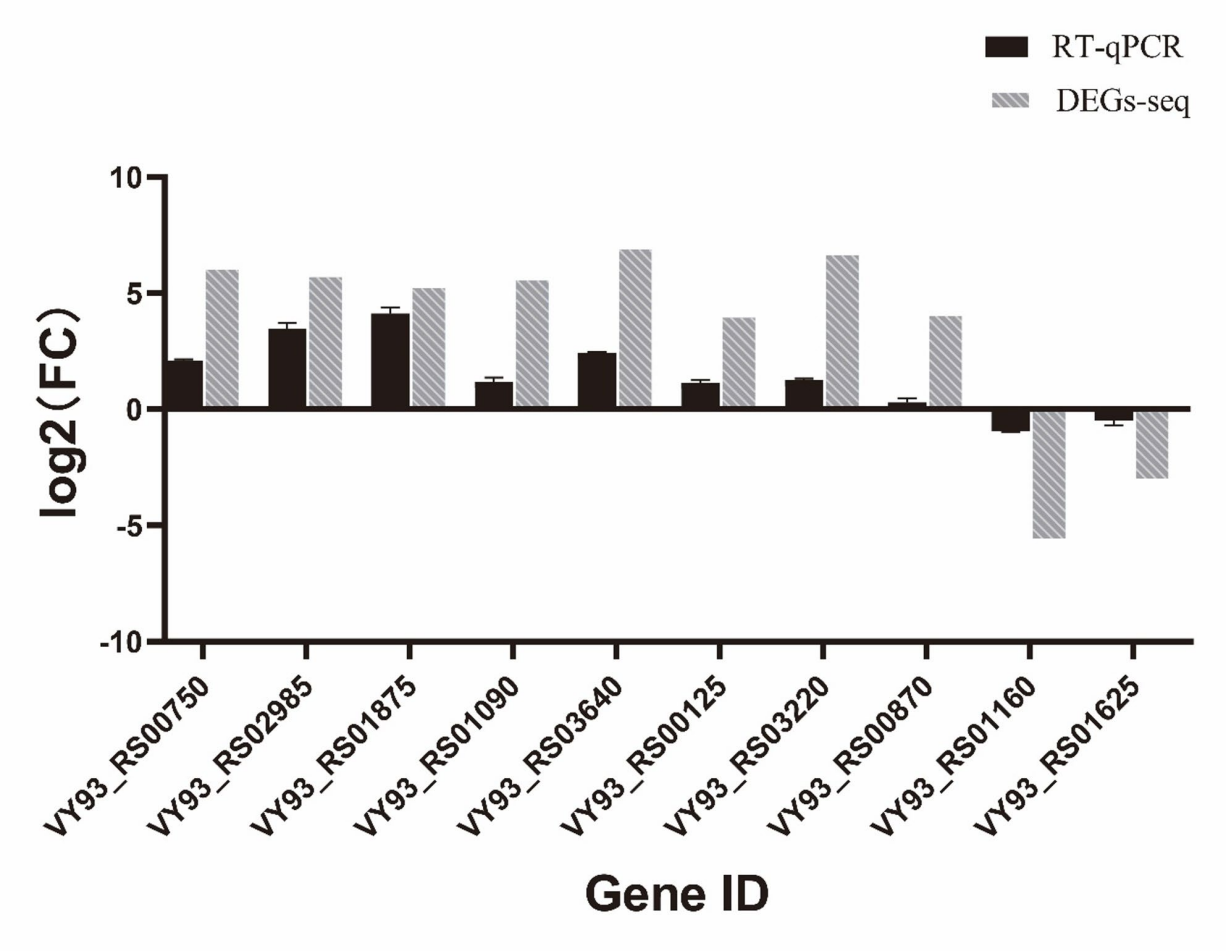
Expression of 10 DEGs as determined by RT-qPCR. The abscissa indicates gene name, the ordinate indicates log2(FC), non-bar columns indicate RT-qPCR values, and bar columns indicate the corresponding gene log2(FC) values via transcriptome sequencing

qRT-PCR outcomes exhibited a congruence with observed trends in up- and downregulation of genes (Fig. 7), indicating that sequence results were reliable.

## Discussion

Numerous instances of interactions between mycoplasmas and the immune system of the host have been substantiated, encompassing the modulation of the host immune system and the provocation of inflammatory responses. These interactions facilitate establishment of chronic and persistent mycoplasma infections [24]. Additionally, mycoplasmas can exert influence on the host organism’s metabolism and physiology by perturbing the process of nutrient absorption, with RNA-seq results indicating that *M. gallisepticum* infection triggers both innate immunity and inflammation in the host’s immune organs [25]. A comparison of chicken embryos and chickens revealed a lower magnitude of immune response-related transcriptional changes in the latter [26] Additionally, a transcriptome analysis of spleen tissues indicated a significant enrichment of DEGs primarily associated with immune processes following infection with *M. synoviae* [27]. Among the four genes we focused on, cysteine protease, neuraminidase and thermonuclease family proteins showed the same expression trend as in previous studies, while hemagglutinins showed the opposite trend and were down-regulated [5]. Interestingly, hemagglutinins, usually acting as adhesins, were down-regulated when exposed to the HD11 immune cell line, unlike other virulence genes in this study. This down-regulation might be a strategy to evade immune detection, meriting further investigation through functional assays.

It is well established that *M. synoviae* infection can lead to systemic infection and/or infectious synovitis in avian species. Infection leads to upregulation of genes associated with phagocytosis and downregulation of genes involved in the host’s protective defense mechanism. These findings offer potential insights into the molecular mechanisms underlying chronic *M. synoviae* infection [6]. Nevertheless, the precise mechanism by which *M. synoviae* infects and persists within the host remains uncertain. Relatively few studies have examined about infection of host cells, and most of those have focused on host changes after mycoplasma infection.

Technological advancements in genome sequencing have resulted in many prokaryote genomes now having been sequenced, which provides valuable insights into these organisms [28, 29]. We sought to elucidate the transcripts expression of *M. synoviae* when it exposed to HD-11 macrophage cells and DF-1 chicken embryo fibroblast cells. Total *M. synoviae* RNA, both exposed to host cells (host cells-challenged) and in *vitro*-cultured, was extracted and used for sequencing. The 5’ UTR is an important region in translation initiation because its length, secondary structure, and presence of AUG codons that influence process efficiency [30]. We identified 83 genes with a 5’ UTR length < 20 nt, which may contribute to leaky scanning. 5’ UTR could be used as a target for small molecules [31], further experiments are required to investigate on *M. synoviae* 5’ UTR, modified 5’ UTR to improve conversion of related therapeutics [32]. sRNA, a substantial portion of bacterial RNA, typically ranges 50–500 nt in length, and plays crucial roles in regulating mRNA stability, transcription, and translation. sRNAs are both essential for post-transcriptional regulation of gene expression in all organisms, and they play critical roles in bacterial virulence regulation [33]. When Listeria parasitizes in animal cells, it produces a long 5 ' -UTR region. The sRNA Rli27 targeting the long 5’-UTR to regulate the cell wall protein Lmo0514 translation level [34]. In bacterial/host interactions, certain bacteria can engage in sRNA-mediated regulation of various virulence determinants, including quorum sensing, type III secretory system (T3SS), iron transport, and biofilm formation [35–37]. We reveal significant upregulation of two sRNAs in *M. synoviae* when exposed to host cells, suggesting their potential involvement in infection. The preliminary association analysis of upregulated sRNA and mRNA provides ideas for future research on sRNA regulation of mRNA translation.

GO enrichment analysis showed that the function of transmembrane transport and related protein complexes was active. Transmembrane transport in organisms involves the transport of solutes through lipid bilayers to realize the metabolism of cellular substances—a basic condition for cell survival [38]. Most membrane proteins (including transport-related enzymes) do not exist independently, but are located in complexes [39]. This study highlights a logical loop involving terms like transmembrane transport, transporter activity, and membrane protein complexes. The notable upregulation of transport-related genes during host cell exposure indicates their metabolic importance in infection, suggesting that these transporters and complexes are promising candidates for future therapeutic or preventive research. The KEGG analysis provides evidence and refinement for this from the specific pathway level, especially the significant enrichment of genes in ABC transporters and biological secretion systems. Among the efflux pumps, ABC transporters have received significant attention because of their association with bacterial resistance [40, 41]. The type III secretion system is of significant importance in the pathogenesis of most Gram-negative bacteria, leading to its exploration as a potential antibiotic target [42]. Furthermore, several studies have identified ABC transporter and bacterial secretion system proteins to be virulence factors in specific microorganisms, suggesting their potential role in pathogenesis and evasion during host infection [43–46]. These secreted effectors, typically virulent factors, play a crucial role in nutrient acquisition, host-cell interaction, and tissue localization specificity [47–51]. We show that many proteins in ABC transporters and secretion systems are upregulated, indicating their possible role in *M. synoviae* infection. This lays the groundwork for future research into their specific functions and potential as intervention targets. This integrated analysis of transcriptomic data expands our current understanding of the metabolic adaptations employed by mycoplasmas during host interaction. However, the characterization of transport and secretion system in mycoplasma remains incomplete, the further investigation is required to determine the specific role of these proteins in the infection process of this mycoplasma. These findings have significant implications for understanding the mechanism of mycoplasma infection [52, 53]. The DEGs were also significantly enriched in the Quorum sensing pathway, which may be related to the ability of *M. synoviae* to form biofilms [54]. Quorum sensing plays a crucial role in regulating bacterial virulence, as well as enhancing antimicrobial resistance, membrane permeability, motility, and biofilm formation [55]. When microbial populations reach a specific density, the molecular concentrations of signaling molecules reach a threshold. This triggers transmission of signals across cells through related proteins, including receptor protein kinases. Consequently, there is an alteration in specific gene expression and the regulation of microbial physiological characteristics [56, 57]. The ability of bacteria to communicate and coordinate activities in the complex environment (i.e., quorum sensing) is crucial for their survival as a population [58]. The up-regulation of quorum sensing-related genes during infection suggests that this pathway may play a role in the infection process, potentially contributing to virulence expression and biofilm formation, which are important factors in the transition from colonization to clinical disease.

Differentially expressed genes in the relevant pathway may be activated in response to the host cell, although the precise mechanism of activation requires further experimental characterization. T The upregulated genes in these pathways offer potential candidates for studying the *M. synoviae* secretion system. Additionally, the two upregulated sRNAs identified could be explored to see if they post-transcriptionally regulate similarly expressed genes. In bacteria, the simultaneous occurrence of DNA replication and transcription, using the same template is crucial for cellular growth, and involves numerous proteins [59]. The antimicrobial potential of proteins involved in DNA replication has been investigated in drug-resistant bacterial pathogens [60–62]. Upon infection of host cells, *M. synoviae* exhibits enhanced DNA replication and transcription, suggesting heightened metabolic activity and stress tolerance during the infection process.

## Conclusions

We characterized the transcriptional landscape of *M. synoviae* under these conditions and predicted the functional components involved in the host-pathogen interaction. Analysis of the signaling pathways and biological functions associated with identified DEGs was performed to elucidate the molecular mechanisms underlying *M. synoviae* persistence. This transcriptomic analysis identifies key pathways, especially in transport and secretion, for further study. Future research should validate the regulatory roles of the identified sRNAs and explore if components of these pathways can be targeted to control *M. synoviae* infection.

## Supplementary Information

Supplementary Material 1.

Supplementary Material 2.

Supplementary Material 3.

Supplementary Material 4.

Supplementary Material 5.

Supplementary Material 6.

## Data Availability

The datasets generated for this study can be found in the NCBI Sequence Read Archive: SRR26081760, SRR26081761, SRR26081762, SRR26081763, SRR26081764, and SRR26081765.
