# Supplementary material for: Transcriptome landscape of Mycoplasma synoviae when exposed to chicken cells
Source: BMC Genomics. 2026 Mar 28;27:449. doi: 10.1186/s12864-026-12748-1 (PMC13151368; doi:10.1186/s12864-026-12748-1)
Supplement: Supplementary file 1 — Supplementary Material 1. [file 12864_2026_12748_MOESM1_ESM.pdf]

RNA Test Report

Samples RNA Testing

1. Pre-check handling (Guideline range for testing concentration: 25-500 ng/μl — Nanodrop value):

a. If the sample concentration according to the Nanodrop value is within the guideline range, proceed directly with Agilent 2100 testing;

b. If the sample concentration according to the Nanodrop value exceeds the guideline range, dilute it to fall within the Agilent 2100 testing range. Multiply the measured value by the dilution factor to obtain the original sample concentration;

c. If the sample Nanodrop concentration is below the guideline range, stop testing and discuss the feasibility of using the Agilent 2100 RNA 6000 Pico kit for detection.

2. Purity and concentration testing

Preliminary quantification — NanoDrop 2000 spectrophotometer test

NanoDrop 2000 blank reference: ddH2O

Accurate concentration quantification — Agilent 2100 RNA 6000 Nano Kit

3. Test results (sample concentration and RIN value determined according to Agilent 2100 results)

Agilent 2100 test results:Sample loading amount: 1 µl of original or diluted sample

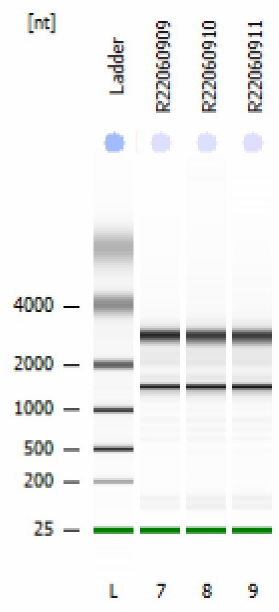

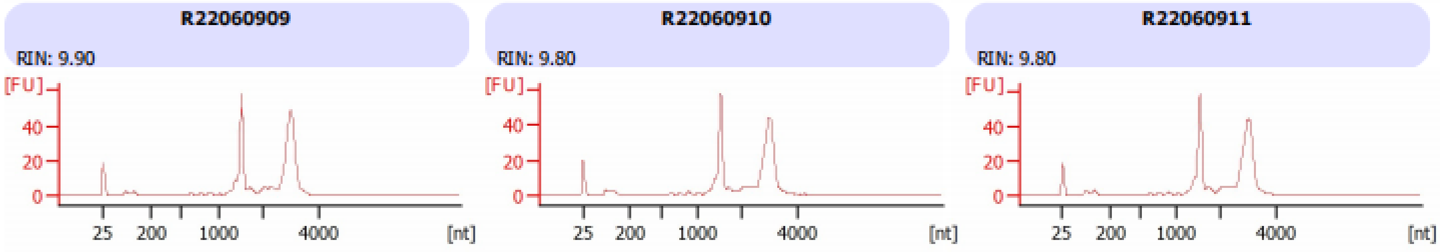

Number	Sample Name	Concentration ng/ul	Volume ul	Total amount ug	RIN	Species	Library Type
R22060909	MS-Host1	186	38	7.07	9.9	Mycoplasma synoviae	lncRNA
R22060910	MS-Host 2	200	33	6.60	9.8	Mycoplasma synoviae	lncRNA
R22060911	MS-Host 3	207	36	7.45	9.8	Mycoplasma synoviae	lncRNA

Controls RNA Testing

The results of testing according to the above method are as follows:

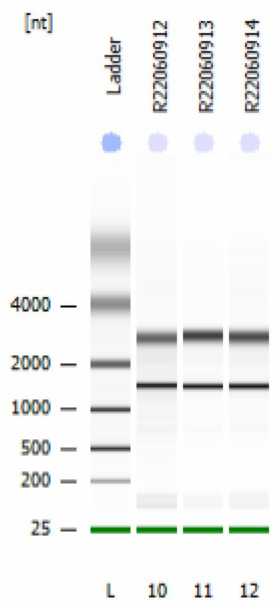

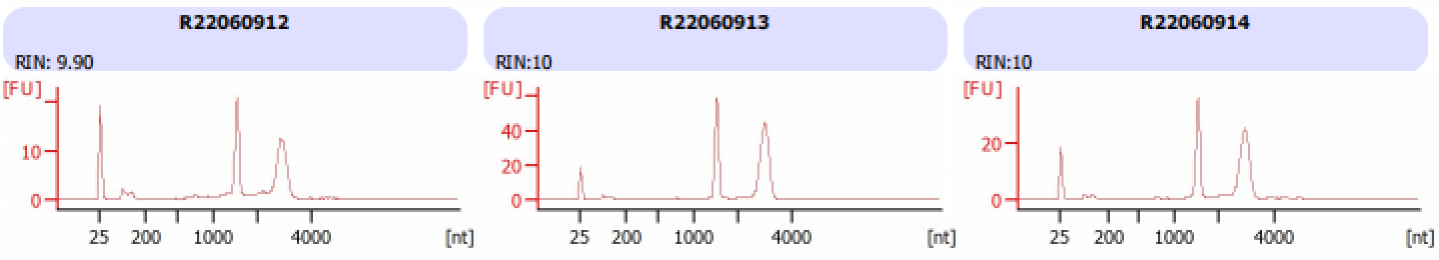

Number	Sample Name	Concentration ng/ul	Volume ul	Total amount ug	RIN	Species	Library Type
R22060912	C1	189	35	6.62	9.9	Mycoplasma synoviae	Bacterial transcriptome
R22060913	C2	353	44	15.53	10	Mycoplasma synoviae	Bacterial transcriptome
R22060914	C3	200	33	6.60	10	Mycoplasma synoviae	Bacterial transcriptome
